# Translation and psychometric testing of the Farsi version of the Seattle angina questionnaire

**DOI:** 10.1186/s12955-017-0808-4

**Published:** 2017-12-02

**Authors:** Zahra Taheri-Kharameh, Majideh Heravi-Karimooi, Nahid Rejeh, Ebrahim Hajizadeh, Mojtaba Vaismoradi, Sherrill Snelgrove, Ali Montazeri

**Affiliations:** 1 School of Paramedical Sciences, Qom University of Medical Sciences, Qom, Iran; 2 Students Research Center, Department of Public Health, Hamadan University of Medical Sciences, Hamadan, Iran; 3 Elderly Care Research Center, Department of Nursing, Faculty of Nursing and Midwifery, Shahed University, Tehran, Iran; 4 Department of Biostatistics, Faculty of Medical Sciences, Tarbiat Modares University, Tehran, Iran; 5 Faculty of Nursing and Health Sciences, Nord University, Bodø, Norway; 6 Mental Health Research Group, Health Metrics Research Centre, Iranian Institute for Health Sciences Research, ACECR, Tehran, Iran; 7 Faculty of Humanity Sciences, University of Science & Culture, ACECR, Tehran, Iran

**Keywords:** Validity, Reliability, Quality of life, Angina pectoris, Farsi version, Seattle angina questionnaire

## Abstract

**Background:**

Angina pectoris causes substantial psychological and functional disabilities and adversely effects quality of life in patients. The aim of this study was to investigate the psychometric properties including validity and reliability of the Farsi version of the Seattle angina questionnaire.

**Methods:**

The ‘forward-backward’ procedure was applied to translate this questionnaire from English to Farsi. The translated version of the Seattle angina questionnaire was assessed in terms of validity and reliability with a convenience sample of 200 patients suffering from angina pectoris who were recruited from the inpatient ward (post CCU) and outpatient department at two teaching hospitals in an urban area of Iran. Validity was assessed using content, face and construct validity. The calculation of the Cronbach’s alpha coefficient and the test-retest method helped with the assessment of reliability of the questionnaire’s five subscales. Construct validity of the questionnaire was evaluated using exploratory factor analysis.

**Results:**

The results of exploratory factor analysis indicated a five-factor solution for the questionnaire including ‘physical limitation in middle to strenuous activities’, ‘physical limitation in slight activities’, ‘angina pattern and discomfort of treatment’, ‘treatment satisfaction’ and ‘disease perception’ that jointly accounted for 64.42% of variance observed. Convergent validity was mostly supported by the pattern of association between the Seattle angina questionnaire-Farsi version and the SF-36. Cronbach’s alpha of the subscales ranged from 0.60 to 0.86 and test-retest scores ranged from 0.79 to 0.97 indicating a good range of reliability.

**Conclusions:**

The Seattle angina questionnaire-Farsi version had acceptable psychometric properties. Therefore, it can be used to assess health-related quality of life and assess the effects of different medical and nursing interventions on patients’ quality of life.

## Background

Cardiovascular disease (CVD) is the leading cause of death and disability across the world [1, 2]. In Iran, a Middle Eastern country, CVD is also the foremost cause of death in adults aged 35 years and older [3]. Angina pectoris is the most common type of CVD and accounts for the majority of hospital admissions. Angina pectoris is characterized by the feelings of chest pressure or pain secondary to imbalance between myocardial oxygen supply and demand caused by the coronary artery obstruction [4]. Patients with angina pectoris usually suffer from fear, anxiety, and impaired ability to perform activities of daily livings [5]. Recurrent episodes of angina also require repeated hospitalization in coronary care units (CCU), which in turn impose stress on patients and family members and heavy financial burdens on the healthcare system [6]. Moreover, angina pectoris threatens patients’ health-related quality of life (QoL). Consequently, monitoring QoL in patients with angina pectoris is very important [7].

Health-related QoL is a state in which patients feel emotionally, socially, and physically satisfied [8]. Patients’ perceptions of a certain medical or surgical treatment are required for judging its success [9]. The evaluation of healthcare outcomes is a fundamental prerequisite for assessing he success of treatment modalities. Different general and disease-specific questionnaires have been developed to evaluate healthcare outcomes such as the patient’s QoL. Disease-specific questionnaires measure healthcare outcomes that are related mainly to the course of the disease and its progression in specific patient populations [7, 10, 11].

These questionnaires deal with the specific aspects of diseases and provide higher response rates due to their relevance for respondents. Moreover, disease-specific questionnaires are more sensitive to health alterations [12, 13].

The Seattle angina questionnaire (SAQ) as a disease-specific questionnaire is developed for the evaluation of health-related QoL in patients with angina pectoris [14]. SAQ has been used extensively in different studies [15–18]. Cultural adaptation and psychometric properties of the original and translated versions of the SAQ have been assessed in different countries including but not limited to USA [19], Japan [20], the UK [21], Norway [22], and Germany [23]. These studies have confirmed that the SAQ is a valid and reliable questionnaire for the measurement of QoL in patients with angina pectoris living in different cultures and contexts. The SAQ was translated into Farsi in a pilot study and early version by Taheri Kharame et al. [24], but they did not evaluate its advance pschomeric properties. Therefore, this study was carried out to translate the SAQ and assess the psychometric properties of the Farsi version of SAQ (SAQ-F) as a disease-specific QoL scale in patients with angina pectoris in Iran.

## Methods

### Participants and study setting

This methodological study was conducted in an urban area in the centre of Iran from June 2014 to March 2015. As the samples of this study, 200 patients were recruited from the inpatient ward (post CCU) and outpatient clinics at two teaching hospitals. The patients were selected using a convenient sampling method according to the following inclusion criteria:

- age more than 20 years;
- being diagnosed with angina pectoris by a cardiologist;
- the history of heart diseases for more than 6 months;
- ability to communicate in Farsi;
- having no previous psychiatric diseases;
- not taking any psychoactive drugs;
- willingness to participate in this study.

The study procedure was explained to those patients who met the eligibility criteria. The questionnaire was given to the patients and analysed by the principal researcher (MHK). The questionnaire completion took between 10 and 15 min.

### Instruments

For data collection, a three-part questionnaire was used as follows:

#### Socio-demographic form

To collect clinical and socio-demographic data of the patients, a questionnaire consisting of questions about age, the marital status, education level, employment, economic status, smoking and medical history was used.

#### SAQ

QoL was measured using the SAQ [14]. It was a disease-specific instrument for patients with CVD. The SAQ-F contained 19 items to quantify five clinically relevant domains of CVD including physical limitations due to the symptoms of angina, angina stability, angina frequency, treatment satisfaction and disease perception. Scores obtained in these domains were transformed and expressed from 0 to 100, where higher scores indicated better quality of life. Since each domain monitored a unique dimension of CVD, no summation of scores was generated.

#### SF-36

QoL was assessed using the SF-36 questionnaire as a general health-related QoL instrument. The SF-36 had eight subscales including physical functioning, bodily pain, general health, vitality, social functioning, role limitations due to physical problems, role limitations due to emotional problems and mental health. Scores in each scale ranged from 0 to 100, with zero representing the worst QoL and 100 representing the best possible score. It was reported that the original and Farsi versions of this questionnaire had appropriate reliability and construct validities [25, 26].

### Translation procedure and evaluation of validity

After obtaining the permission from the author for the translation and application of the SAQ, it was translated as a part of the international quality of life assessment project (IQoLAP) [25]. This  approach to translation and validation has been developed for use with the SF-36, but it is also applicable to other translation efforts. The questionnaire was translated from English to Farsi by two doctoral nurses and the primary Farsi version of the questionnaire was developed based on the comparison of the two translations. Next, the Farsi version was back-translated to English by a translator who had no previous knowledge of the SAQ. The original and back-translated versions were compared item by item and a final Farsi version of the questionnaire was reached. After the completion of translation, a multidisciplinary panel of healthcare professionals and academics was developed to test content validity of the questionnaire. The panel included two cardiologists, six nursing professionals as faculty of members of two medical sciences universities in Iran, and two clinical nurses with the experience of working in the cardiology ward. They were asked to comment on the reasonability and suitability, and logical sequence of items as well as comprehensiveness of the questionnaire. Moreover, to assess the questionnaire’s face validity, it was given to 10 patients with angina pectoris to assess its comprehensibility and legibility. According to the presented comments and perspectives by the experts and patients, some items were modified. The instrument was anglicized by slight wording changes of three items: (i), within the physical limitations dimension the phrase of “walking more than one block.. .” was changed to “walking more than 100 metres.. .”, (ii), the phrase “past 4 weeks” was changed to “past 1 month” within 3, 4 and 9 items, and (iii), the word “bothersome” was changed to “worrisome” for one of the items within the treatment satisfaction dimension.

For data collection, the study procedure was explained to the patients who met the eligibility criteria. The questionnaire’s items were read for those patients who were unable to read it and their answers were checked by the principal researcher. The questionnaire completion took between 10 and 15 min.

Two weeks after that the first survey, the SAQ-F was disseminated again to 30 patients who had responded to the first set of questionnaires and who had agreed to fill in the SAQ-F administered twice-with a two-week interval [27]. This procedure was conducted to check the questionnaire reliability using the test-retest method.

### Data analysis

Data analysis was conducted using SPSS v.16 software for Windows. Patient characteristics and the score for each domain of the SAQ-F were analysed by using descriptive statistics. Construct validity of the questionnaire was performed using exploratory factor analysis. Principle components analysis with varimax rotation was applied. The Kaiser-Meyer-Olkin (KMO) and Bartlett’s Test of Sphericity were used to assess the appropriateness of the sample for factor analysis. Eigen values above 1 and the scree plot were used to determine the number of factors. Factor loadings equal or greater than 0.4 were considered appropriate [28]. As known group comparison, the SAQ-F scores of patients with and without chest symptoms were evaluated using the Mann-Whitney U test. To assess the concurrent validity of the SAQ-F, the Pearson’s correlation coefficient between the scores of the SAQ-F and SF-36 was computed. Internal consistency of each scale of the SAQ-F was determined using the calculation of the Cronbach’s alpha coefficient. Cronbach’s α coefficient 0.7 or above was considered to be satisfactory [29]. Test-retest reliability was assessed by computing the intra-class correlation coefficient of each domain. The time interval for this assessment was 2 weeks in this study. An ICC > 0.80 indicated good test–retest reliability and stability [28].

## Results

### The demographic characteristics of the patients

Mean (standard deviation) of the patients’ age was 59.46 years (SD = 11.24). Also, 57.5% were females and 84.5% were married. Further information about the characteristics of the participants were presented in Table 1.

**Table 1:** The clinical and socio-demographic information of the patients (*N* = 200)

	Number	Percent
Age (years)		
Mean (SD)	59.46	11.24
Gender		
Male	85	42.5
Female	115	57.5
Education level		
Illiterate	81	40.5
Primary school	87	43.5
High school	25	12.5
Secondary school	7	3.5
Marital status		
Single	2	1
Married	169	84.5
Divorced/widow	29	14.5
Employment status		
Employed	60	30
Unemployed/housewife	140	70
Economic status		
Poor	93	46.5
Good	107	53.5
Smoking status		
Smoker	51	25.5
Non-smoker	149	74.5
Family history of CVD	81	40.5
Yes	88	92.63
No	7	3.37
Duration of heart disease, y		
Mean (SD)	3.47	3.37
Medical history		
Hypertension	103	51.5
Diabetes mellitus	78	39
Hypercholesterolaemia	95	47.5
Previous cardiac revascularizaton	48	24
Angina functional class		
I	59	29.5
II	89	44.5
III	50	25
IV	2	1
Ejection fraction (%)		
Mean (SD)	53	4.6

I: no limitation of ordinary physical activity

II: slight limitation of ordinary physical activity

III: marked limitation of ordinary physical activity

IV: unable to carry on any physical activity without discomfort

### Construct validity

#### Exploratory factor analysis

**Fig. 1:**
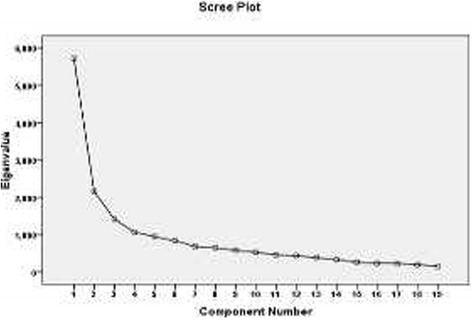
A scree plot illustrating the factor loading of the SAQ-F

The Kaiser-Meyer-Olkin adequacy was 0.80 and Bartlett’s test of sphericity was significant (*p* < 0.001), indicating sample adequacy. Factor analysis with principal component factor analysis and varimax rotation was used to determine construct validity by excluding items with factor loadings below 0.3. After varimax rotation, a total of 19 items loaded significantly on five factors. All five factors had an eigenvalue greater than 1, with an explained variance of 64.42%. Except one item, factor loadings ranged from 0.53 to 0.85. One item was deleted (factor loading <0.3). The scree plot suggested generating a five-factor model (Fig. 1).

The first factor was related to physical limitation in middle to strenuous activities (4, 5, 6, 7, 8, 9); The second factor was physical limitation in slight activities (1, 2, 3); The third factor was angina pattern and discomfort of treatment (11, 12, 13); The fourth factor was treatment satisfaction (14, 15, 16); The fifth factor was disease perception (17, 18, 19). Further information about the factor structure was reported in Table 2.

**Table 2:** Principal component analysis of the SAQ-F

Item	Factor1	Factor2	Factor3	Factor4	Factor5
Physical limitation in middle to strenuous activities					
Climbing a hill/stairs	0.603				
Gardening, etc	0.745				
Walking 100 m or more	0.807				
Lifting heavy objects	0.855				
Running or jogging	0.800				
Strenuous sports	0.603				
Physical limitation in slight activities					
Dressing		0.789			
Walking indoors		0.778			
Showering		0.797			
Angina pattern and discomfort of treatment					
Frequency of symptoms			0.615		
Use of tablets			0.723		
Troublesome and pills			0.720		
Treatment satisfaction					
Satisfaction that everything is done				0.831	
Satisfaction with doctor’s explanation				0.772	
Overall satisfaction with treatment				0.765	
Disease perception					
Interference with life enjoyment					0.534
Feelings about symptom persistence					0.791
Worry about heart attack/death					0.710

#### Discriminant validity

With regard to the known group comparison (Table 3), the patients without chest symptoms exhibited significantly higher SAQ-F scores than those who had poor scores for the physical limitation in slight activities, angina pattern and discomfort of treatment and disease perception domains.

**Table 3:** The known-group comparison of the SAQ-F

Domain score	Asymptomatic M(SD) *n* = 30	Symptomatic M(SD) *n* = 170	*P*-value
Physical limitation in middle to strenuous activities	34.85 (39.86)	25.71 (35.21)	0.868
Physical limitation in slight activities	16.22 (91.66)	21.19 (84.62)	0.023
Angina pattern and discomfort of treatment	8.82 (96.33)	18.84 (63.91)	0.0001
Treatment satisfaction	20.30 (65.24)	20.46 (60.12)	0.219
Disease perception	24.26 (60.34)	21.19 (35.36)	0.0001

#### Convergent validity

Table 4 showed the correlation between SAQ-F and SF-36, which was used to assess convergent validity. SAQ-F score demonstrated a significant and small-to-moderate levels of correlation with the SF-36 score (*r*_=_ 0.17– 0.69, *p* < 0.01).

**Table 4:** The correlation between the SAQ-F and SF-36

Variable	Physical limitation in middle to strenuous activities	Physical limitation in slight activities	Angina pattern and discomfort of treatment	Treatment satisfaction	Disease perception
Physical function	0.520^**^	0.691^**^	0.379^**^	0.174^**^	0.359^**^
Role physical	0.245^**^	0.361^*^	0.328^**^	0.208^**^	0.206^**^
Bodily pain	0.374^**^	0.415^**^	0.393^**^	0.241^**^	0.516^**^
General health	0.172^*^	0.415^**^	0.391^**^	0.240^**^	0.405^**^
Vitality	0.185^**^	0.338^**^	0.361^**^	0.220^**^	0.350^**^
Social function	0.351^**^	0.353^**^	0.381^**^	0.248^**^	0.395^**^
Role emotional	0.240^**^	0.318^**^	0.335^**^	0.191^**^	0.260^**^
Mental health	0.188^*^	0.269^**^	0.241^**^	0.220^**^	0.250^**^

**Correlation is significant at the 0.01 level

*Correlation is significant at the 0.05 level

### Reliability

Cronbach coefficients for the subscales ranged between 0.54 and 0.88. For the test–retest reliability, the ICC coefficients ranged between 0.79 and 0.97 (*p* < 0.001). Table 5 showed internal consistency and test retest reliability of the SAQ-F.

**Table 5:** Reliability of the SAQ-F

Domain score	Item number		ICC	*P*-value
Physical limitation in middle to strenuous activities	6	0.869	0.91	0.001
Physical limitation in slight activities	3	0.778	0.97	0.001
Angina pattern and discomfort of treatment	3	0.602	0.79	0.001
Treatment satisfaction	3	0.740	0.90	0.001
Disease perception	3	0.661	0.91	0.001

## Discussion

The aim of this study was to assess the psychometric properties of the SAQ-F with an Iranian population. Previous studies across cultures were conducted to assess and promote QoL in patients with CVD[13]. Most of these studies aimed to investigate the effects of nursing interventions on QoL in patients with CVD. However, an absolute prerequisite of these studies is the availability of a standard, valid and reliable questionnaire.

In this study, the SAQ-F was translated based on the instrument translation and cultural adaptation guidelines [30]. The strengths of this study were the four steps of instrument translation and ensuring the cultural adaptation of the translated version. Face and the content validities of the questionnaire were confirmed after some minor revisions.

Moreover, the exploratory factor analysis was used to evaluate construct validity of this questionnaire. The KMO value was high and the Bartlett’s test was significant indicating the appropriateness of the factor analysis model. The scree plot showed that the SAQ-F was consisted of five factors. These factors included ‘physical limitation in middle to strenuous activities’, ‘physical limitation in slight activities’, ‘angina pattern and discomfort of treatment’, ‘treatment satisfaction’ and ‘disease perception’. However, this factor structure was not identical to the original structure [14]. Applying the SAQ-F in different cultures potentially results in discrepancies in the factor structure. The difference between the original and the SAQ-F was that the ‘anginal stability’ subscale was deleted from the current Farsi version. Given the fact that this subscale was consisted of only one item, its deletion could be justifiable. Moreover, in the Farsi version, the ‘physical limitation’ subscale of the original version was divided into two subscales including ‘physical limitation in middle to strenuous activities’ (consisting of six items) and ‘physical limitation in slight activities’ (consisting of three items). Similarly, Kimble et al. [31] reported the division of physical limitation subscale in two separate factors including ‘limitation in activities with middle to high exertional requirements’ and ‘limitation in activities with low exertional requirements’ in women with chronic stable angina.

The factor structure of the SAQ-F as determined in the current study was consisted of five factors. As previously indicated, while they were not identical to the original version, the SAQ-F also reported a five-factor structure [14]. In comparison, Garratt et al. [21] evaluated the psychometric characteristics of the British version of the SAQ and reported a structure consisting of three factors including treatment satisfaction, angina frequency and perception and physical limitations. Kimble [31] also evaluated the psychometric properties of the SAQ in a sample of 175 women with chronic stable angina and reported a six-factor structure.

In this study, a known-groups comparison method to evaluate the discriminant validity of the SAQ-F was conducted. Accordingly, the questionnaire was administered to a sample of symptomatic and asymptomatic patients. The study findings revealed that QoL scores in asymptomatic patients in three dimensions of the SAQ-F including ‘physical limitation in activities requiring lower levels of exertion’, ‘angina pattern and discomfort of treatment’, and ‘disease perception’ were significantly higher than the symptomatic patients. Seki et al. [20] also reported significantly higher QoL scores in four out of five dimensions of the Japanese version of SAQ in asymptomatic patients—the only exception was related to the ‘anginal stability’. Nishiyama et al. [32] also reported that asymptomatic patients obtained significantly higher QoL scores compared to symptomatic patients.

To establish the criterion-related validity of the SAQ-F, the SAQ and SF-36 QoL questionnaires were administered to the participants. The study findings revealed a significant small-to-moderate levels of correlation between all the subscales of the two questionnaires with correlation coefficients ranged from 0.17 to 0.69. Previous studies also reported the same findings [20–22].

The study findings also revealed that the SAQ-F had an acceptable internal consistency. Coronbach’s alpha for different subscales of the SAQ-F ranged between 0.60 and 0.86. Two subscales including ‘angina pattern and discomfort of treatment’ and ‘disease perception’ had a Coronbach’s alpha coefficient less than 0.70. This may be attributed to the small number of items in these two subscales. Waltz et al. [33] noted that the number of items of a measure directly contributed to the magnitude of its Coronbach’s alpha coefficient. The smallest Coronbach’s alpha coefficient in this study was related to the ‘angina pattern and discomfort of treatment’ subscale reported as 0.60. Garratt et al. [21] assessed validity and reliability of the English version of the SAQ in a sample of 655 patients with CVD and reported Coronbach’s alpha coefficients of 0.83–0.92. Coronbach’s alpha coefficients in a study conducted by Seki et al. [20] were between 0.51 and 0.96 with 331 patients who suffered from CVD. Pettersen et al. [22] also evaluated validity and reliability of the Norwegian SAQ and reported Coronbach’s alpha coefficients between 0.70 and 0.92 with 885 patients who suffered from prior myocardial infarction.

In this study, reliability of SAQ-F was evaluated by using the test-retest method. Accordingly, the SAQ-F was assessed to the study participants twice—with a two-week interval. The lowest ICC coefficients were related to the ‘angina pattern and discomfort of treatment’ subscales, respectively. This finding could be attributed to the high recurrence rate of angina episodes as well as to the fact that the study sample was consisted of both hospitalized patients and patients referred to outpatient care settings. Compared to the original version, the Farsi version had better results with regard to the domains. Garratt et al. [21] also reported an acceptable range of stability between 0.63 and 0.81 for the UK version of the SAQ [21]. However, Pettersen et al. [22] reported ICC values between 0.29 and 0.84. Seki et al. [20] in their study found ICC values between 0.41 and 0.79 that were below the recommended values (ICC < .80).

## Conclusions

The results of this study showed that the SAQ-F had acceptable psychometric properties. This questionnaire can be used to measure healthcare outcomes in different clinical settings and research centres across the world. The SAQ-F is easy to understand and respond and takes less than 10 min to be completed. It is noteworthy that the SAQ-F is used for both measuring health-related QoL and assessing the effects of different medical and nursing interventions on patients’ QoL.

### Limitations of study

Non-random sampling restricts the generalizability of the study findings. Consequently, multi-centre or multi-state studies with larger sample sizes are recommended. Moreover, since we evaluated only the validity and the reliability of the SAQ-F, more studies for assessing the responsiveness of the SAQ are also recommended. Finally, further development of culturally appropriate QoL questionnaires using qualitative studies is another potential area of study.

## Abbreviations

CVD: Cardiovascular disease
QoL: Quality of life
SAQ: Seattle angina questionnaire
SAQ-F: Farsi version of SAQ
